# Different Types of Long-Term Milk Consumption and Mortality in Adults with Cardiovascular Disease: A Population-Based Study in 7236 Australian Adults over 8.4 Years

**DOI:** 10.3390/nu14030704

**Published:** 2022-02-08

**Authors:** Xiaoyue Xu, Alamgir Kabir, Margo L. Barr, Aletta E. Schutte

**Affiliations:** 1 School of Population Health, University of New South Wales, Sydney 2052, Australia; a.schutte@unsw.edu.au; 2 Cardiovascular Division, The George Institute for Global Health, Sydney 2042, Australia; 3 Centre for Primary Health Care and Equity, University of New South Wales, Sydney 2052, Australia; a.kabir@unsw.edu.au (A.K.); margo.barr@unsw.edu.au (M.L.B.); 4 Hypertension in Africa Research Team, Medical Research Council Unit for Hypertension and Cardiovascular Disease, North-West University, Potchefstroom 2520, South Africa

**Keywords:** milk, dairy, cardiovascular health, longitudinal, death, sex differences, secondary prevention

## Abstract

Most studies disregard long-term dairy consumption behaviour and how it relates to mortality. We examined four different types of long-term milk consumption, namely whole milk, reduced fat milk, skim milk and soy milk, in relation to mortality among adults diagnosed with cardiovascular disease (CVD). A retrospective population-based study was conducted in Australia (the 45 and Up Study) linking baseline (2006–2009) and follow-up data (2012–2015) to hospitalisation and mortality data up to 30 September 2018. A total of 1,101 deaths occurred among 7236 participants with CVD over a mean follow-up of 8.4 years. Males (Hazard Ratio, HR = 0.69, 95% CI (0.54; 0.89)) and females (HR = 0.59 (0.38; 0.91)) with long-term reduced fat milk consumption had the lowest risk of mortality compared to counterparts with long-term whole milk consumption. Among participants with ischemic heart disease, males with a long-term reduced fat milk consumption had the lowest risk of mortality (HR = 0.63, 95% CI: 0.43; 0.92). We conclude that among males and females with CVD, those who often consume reduced fat milk over the long-term present with a 31–41% lower risk of mortality than those who often consume whole milk, supporting dairy advice from the Heart Foundation of replacing whole milk with reduced fat milk to achieve better health.

## 1. Introduction

Dairy consumption, with milk as the most basic dairy product, is a bone of contention in terms of overall health and cardio-protection. The recommendation of dairy intake included in healthy dietary guidelines vary from region to region, but overall, most countries recommend including dairy as part of a healthy diet because it is rich in calcium and essential nutrients [1,2]. Guidelines generally recommend a daily dairy intake between 2 and 3 servings of milk, or yogurt or cheese for adults [3].

Originally the common understanding was that full-fat dairy products contribute to the intake of saturated fats, which may lead to increased low-density lipoprotein cholesterol levels, and thus has long been considered a risk factor for cardiovascular disease (CVD) [4,5]. This view was incorporated into healthy dietary guidelines which often recommended people to choose dairy in fat-free or low-fat forms. For example, the American Heart Association recommends that adults consume 2–3 servings of fat-free or low-fat dairy products per day [2]. The Eating Well Guideline from the United Kingdom suggests that men and women consume 3 portions of dairy products per day, specifically low-fat options [6].

However, in recent years, observational studies and meta-analyses found contradictory results with respect to the different types of dairy or milk (e.g., whole milk and reduced fat milk) in relation to health, particularly in terms of total mortality and cardiovascular mortality [4,7,8]. Some studies concluded that full-fat dairy is not clearly associated with risk of CVD [9], with some indicating that full-fat dairy plays a protective role in the prevention of CVD [10,11,12]. This protection is explained by the high bioavailability of high-value nutrients and anti-inflammatory properties of full-fat dairy [13], including specific amino acids, medium-chain and odd-chain saturated fats, phospholipids, unsaturated and branched-chain fats, natural trans fats, vitamin K1 and K2 and calcium [8,14,15]. Therefore, some leading cardiovascular organisations such as the American College of Cardiology [16] and European Society of Cardiology [17] suggest a reconsideration of the recommendation on dairy consumption, in particular the role of different types of dairy in cardio-protection. These recommendations are not only relevant to the broader population as part of primary prevention, but especially in those who were already diagnosed with CVD and as a concrete part of secondary prevention.

In addition, important aspects regarding dairy intake were not thoroughly investigated in previous studies. Firstly, limited studies reviewed long-term dairy or milk consumption in relation to cardiovascular mortality, with most studies commonly linking a single dairy or milk consumption report to mortality outcomes [5]. Secondly, the role of dairy products in the secondary prevention and management of CVD is largely overlooked; hence, attention needs to be directed to the critical part that diet can play to prevent cardiovascular events. Most studies focus on dairy consumption and the risk of incident CVD as part of primary prevention (10–12), with very few tracking dairy consumptions among people diagnosed with CVD. We therefore focused on people diagnosed with CVD. Thirdly, limited studies distinguished between different types of milk and how these relate to mortality [7] or reviewed sex differences, as it is known that there are sex-specific food choice preferences for energy and nutrient intake [18].

To fill these research gaps, we examined whether different types of long-term milk consumption, including whole milk, reduced fat milk, skim milk and soy milk, are related to mortality among adults diagnosed with CVD by using Australia’s largest ongoing study of health and ageing. Given the importance of sex-specific features in CVD, we performed all analysis stratified by sex.

## 2. Materials and Methods

### 2.1. Data Sources

This research used baseline (2006–2009) and follow-up data (2012–2016) from the Sax Institute’s 45 and Up Study [19] linked to hospitalisations and mortality data (up to 30 September 2018) by the New South Wales (NSW) Centre for Health Record Linkage (CHeReL) using probabilistic linkage [20].

The 45 and Up Study is a large-scale Australian cohort study focusing on the population of the state of NSW, Australia. Prospective participants were randomly sampled from Services Australia (formerly the Australian Government Department of Human Services) Medicare enrolment database, which provides near complete coverage of the population. People 80+ years of age and residents of rural and remote areas were oversampled. About 18% of those invited participated, and participants included about 11% of the NSW population aged 45 years and over. A total of 267,153 participants joined the study by completing a baseline questionnaire (between January 2006 and December 2009) and giving signed consent for follow-up and linkage of their information to routine health databases. The first follow-up survey data were collected between 2012 and 2015. At both time points, socioeconomic, health behaviour and health-related information were collected via a comprehensive questionnaire. Details of the 45 and Up Study, including sampling strategy and methods, are described elsewhere [21].

Data from study participants were linked to the NSW Admitted Patient Care Data Collection (APDC), providing all public and private hospital admissions in NSW (2001–2018). It contains details of participant admission, dates of admission and discharge, the primary reason for admission, up to 55 additional clinical diagnoses using the World Health Organization International Classification of Diseases, 10th—Australian Modification (ICD-10-AM) and up to 59 operations or procedures [22]. Vital status and date of death were ascertained from the date of recruitment up to 30 September 2018 using linkage to the NSW Registry of Births, Deaths and Marriages. Death registrations capture all deaths in NSW. Cause of death information was only available until 30 June 2016 at the time of analysis and was not included in the analysis.

### 2.2. Ethics

Ethical approval was granted for this research by the New South Wales Population and Health Services Research Ethics Committee (Reference Number: 2016/06/642) and from the University of New South Wales Human Research Ethics Committee (HREC) for the 45 and Up Study.

All participants provided written consent before participating in the 45 and Up Study which included consent to: follow them over time using their health and other records, contact them in the future about changes in health and lifestyle, use their data for health research.

### 2.3. Participant Cohort

For this study, the cohort includes participants diagnosed with CVD aged 45 and over, which was defined as the first hospitalisation following recruitment into the 45 and Up Study with a diagnosis of CVD at discharge based on the ICD-10-AM three-character codes of I00-I99, G45, and G46 [22].

**Figure 1:**
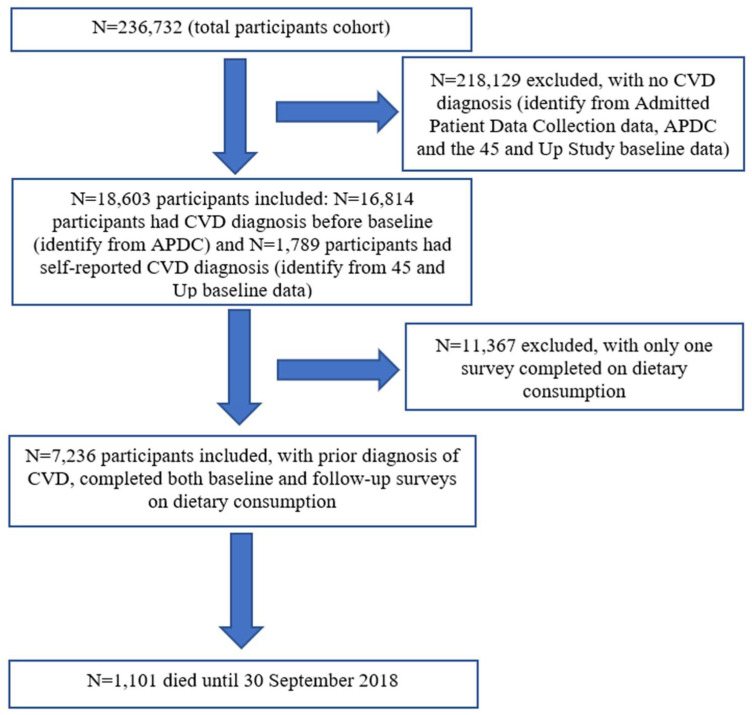
Study flow-chart.

We included participants diagnosed with CVD (identified from APDC data) before the 45 and Up baseline survey, and who completed both baseline (2006–2009) and follow-up surveys (2012–2016) on dietary consumption. Participants who self-reported heart disease at the 45 and Up Study baseline survey were also included. Participants were followed until their date of death (according to the latest available mortality data) or the end of follow-up, whichever came first. The study flow-chart is shown in Figure 1.

#### 2.3.1. CVD Diagnosis

We included males and females with diagnosed CVD, including eight categories of CVD generated from major CVD codes during hospitalisation by making use of routinely collected data (23). The eight categories of CVD include: (1) hypertensive diseases, (2) ischaemic heart disease (IHD), (3) pulmonary heart disease and diseases of pulmonary circulation, (4) other forms of heart disease, (5) cerebrovascular disease, (6) diseases of arteries, arterioles and capillaries, (7) diseases of veins, lymphatic vessels and lymph nodes and (8) episodic and paroxysmal disorders. The details of these major CVD categories are described elsewhere [23].

#### 2.3.2. Dairy Consumption

In the 45 and Up questionnaire, dietary consumption was assessed using short food frequency questions, which were described in our previous studies [24,25]. Each of the questions on diet was validated and applied for in different studies [24,25,26].

We identified four types of milk consumption: whole milk, reduced fat milk, skim milk and soy milk based on the questions: “which type of milk do you mostly have?” Different types of long-term milk consumption were identified for participants who had complete data on consistent milk consumption behaviour at two-time points (baseline and follow-up). For instance, if participants reported to consume whole milk at baseline and follow-up, we considered this behaviour as having long-term whole milk consumption. Milk consumption behaviour was collected across two data collection points over a mean follow-up of 8.4 years.

#### 2.3.3. Co-Variates

We included socio-demographic factors, health behavioural factors, consumption of other food groups and some other self-reported chronic conditions as covariates in the statistical analysis.

Socio-demographic variables included age, marital status, education and socioeconomic level. Marital status was categorised as married/partner, single/divorce/separated and widowed. Education levels were divided into three categories: Low, no school certificate or other qualification, and school, or intermediate certificate; Medium, high school or leaving certificate, and trade or apprenticeship; and High: certificate or diploma, and university degree or higher. Socioeconomic levels were assessed by Socio-Economic Indexes For Areas (SEIFA), which is based on three quantiles (low, medium, high) of the Index of Relative Socio-economic Advantage and Disadvantage [27].

Health behaviours included smoking, alcohol drinking and physical activity levels. Smoking was identified as never smoked, previous smoker and current smoker, based on two questions: “Have you ever been a regular smoker?” and “Are you a regular smoker now?”. The frequency of alcohol consumption was identified by a question of “about how many alcoholic drinks do you have each week?”. Physical activity was measured using the Active Australia Survey, asking the total time spent on walking, and on moderate-intensity and vigorous-intensity physical activity in the previous week. Adequate physical activity was identified if people spent 150 min of moderate intensity physical activity or 75 min of vigorous intensity physical activity per week [28].

Based on the Australian dietary guidelines [1], other food components were also included as covariates, namely (1) vegetables, (2) fruit, (3) grains, (4) lean meat and poultry [24]. The frequency of consuming these food groups was also asked in the 45 and Up dietary questionnaire. We also included self-reported hypertension and diabetes as covariates given the strong association of these conditions with CVD (24). These details are described in our previous publications [24,25].

### 2.4. Statistical Analysis

The *N* (%) for categorical variables and mean (SD) for continuous variables were used to present baseline characteristics by four different types of long-term milk consumption behaviours with sex-specific data representation for males and females. The chi-square test was used to compare statistical differences between categorical variables, and ANOVA was applied to examine statistical differences of means of a continuous variable (e.g., servings of fruit consumption per day) for each category of milk consumption.

A time-to-event analysis was carried out to measure the impact of different types of milk consumption on mortality (*N* = 7236). The follow-up time started at the baseline interview date and censored at death, or 30 September 2018, which ever came first. Kaplan–Meier survival curves for the four categories of milk consumption were generated and a log-rank test was performed to compare the categories. Crude and adjusted hazard ratios (HRs) and their 95% confidence intervals (CIs) were estimated using univariate and multivariable Cox proportional hazard regression models by exponentiating the coefficients of the model and their 95% CIs. The potential confounders, including socio-economic factors (age, marital status, education and socioeconomic level), health behaviours (smoking, alcohol drinking and physical activity levels), consumption of other food groups (vegetables, fruit, grains, lean meat and poultry) and other chronic conditions (hypertension and diabetes), were included in the multivariable Cox model for adjustment.

We also present findings by performing mixed effect models for repeat measurements to test whether the four different types of long-term milk consumption affect the consumption of other foods over time. Coefficients and 95% CI in both the crude and adjusted model were presented. The detailed method is described in our previous study [25]. All analyses were conducted in STATA/SE 14 (STATA, StataCorp, College Station, TX, USA).

### 2.5. Sensitivity Analyses

Since participants with different milk consumption behaviours may die before completing the follow-up survey or by simply not completing the follow-up survey, we performed a sensitivity analysis by only linking baseline dairy consumption data with mortality (*N* = 18,693).

Kaplan–Meier survival estimates and Cox proportional hazard regression models were also applied to examine the different types of milk consumption and mortality in the sensitivity analysis (Supplementary File). We presented the HR (95% CI) in crude models and models with adjustments with baseline socio-economic status, health behaviours and the consumption of other food groups.

## 3. Results

A total of 7236 participants were included in the analysis. The mean follow-up time from recruitment of this cohort is 8.4 years and 4.0 years for mortality, corresponding to 29,185 person-years and 1101 deaths until 30 September 2018. Baseline characteristics according to different types of long-term milk consumption for males and females are shown in Table 1. There were 48.1% of males and 53.6% of females that commonly consumed reduced fat milk followed by whole milk (40.3% for males and 31.0% for females). Differences were found according to four types of milk consumed (namely, whole, reduced fat, skim and soy milk) and age, marital status, socioeconomic levels, smoking status and physical activity. Males with long-term whole milk consumption had higher alcohol drinking than those with other types of milk consumption (*p* < 0.001).

Significant differences were also found across the four types of milk consumption and consumptions of vegetables (*p* = 0.005), fruit (*p* < 0.001), grain (*p* < 0.001) and protein (*p* = 0.002) in males, and consumption of fruit (*p* < 0.001) and grains (*p* < 0.001) in females (Table 1).

For both males and females, we found differences according to four types of long-term milk consumption and survival status, with the smallest proportion surviving consuming whole milk for males and females (both *p* < 0.001) (Table 1).

**Figure 2:**
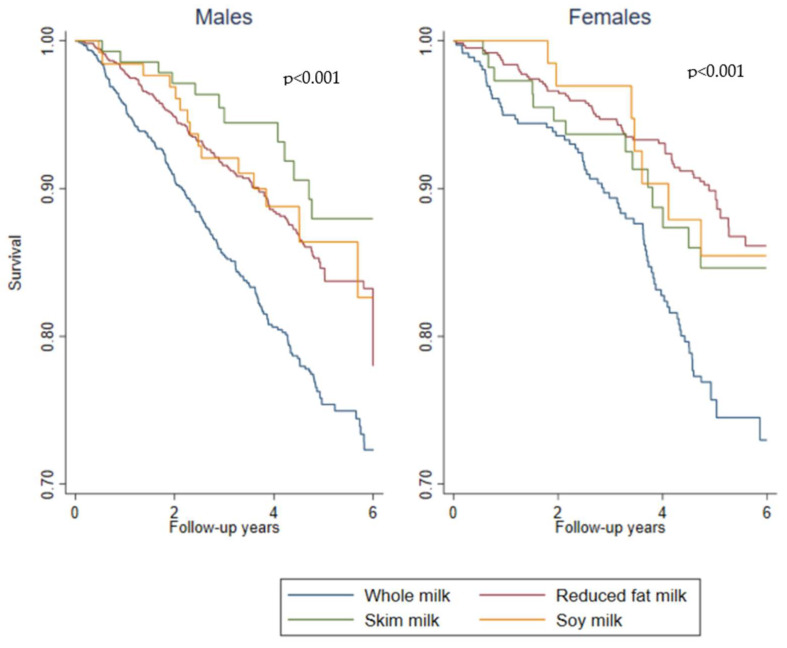
Kaplan–Meier survival estimates for different types of long-term milk consumption and survival by males and females.

Kaplan–Meier survival estimates for different types of long-term milk consumption and survival are shown in Figure 2. Clear differences were found across different types of long-term milk consumption and survival for males and females (both *p* < 0.001), with whole milk having the lowest survival rates and skim milk (males) and reduced fat (females) with the highest survival rates.

Crude and adjusted HR and 95% CI in terms of the association between different types of long-term milk consumption and survival status are shown in Table 2. We tested these associations for males and females with CVD, as well as for each category of CVD (described in the method). Among males and females with CVD, in the crude model, compared with long-term whole milk consumption, males (HR = 0.58, 95% CI: 0.47; 0.72) and females (HR = 0.44, 95% CI: 0.31; 0.62) with long-term reduced fat milk consumption had a lower risk of mortality; males with long-term skim milk (HR = 0.40, 95% CI: 0.22; 0.71) and soy milk (HR = 0.55, 95% CI: 0.33; 0.92) also had a lower risk of mortality. In the adjusted mode, males (HR = 0.69, 95% CI: 0.54; 0.89) and females (HR = 0.59, 95% CI: 0.38; 0.91) with long-term reduced fat milk consumption had the lowest risk of mortality.

We also found that different types of long-term milk consumption were significantly associated with survival among participants with IHD in males (*N* = 3424, Table 2). Compared to males with a long-term whole milk consumption, males with a long-term reduced fat milk consumption had the lowest risk of mortality in the adjusted model (HR = 0.63, 95% CI: 0.43; 0.92). This was, however, not found for females. In addition, no significant associations were found between different types of long-term milk consumption and survival for other types of CVD.

We further tested whether different types of long-term milk consumption affect the consumption of other foods. After adjusting for co-variates, no associations were found between different types of long-term milk consumption and the specific food groups, namely vegetable and protein, but we found strong associations between milk, grain and fruit intake in males and females. Compared to participants with whole milk consumption, males with other types of milk (*p* < 0.001), and females with reduced fat milk (coefficient = 0.86, 95% CI: 0.54; 1.18) and skim milk (coefficient = 0.74, 95% CI: 0.22; 1.26) had higher grain consumption. Males with reduced fat milk (coefficient = 0.11, 95% CI: 0.001; 0.23), skim milk (coefficient = 0.40, 95% CI: 0.18; 0.63) and soy milk (coefficient = 0.57, 95% CI: 0.34; 0.81), and females with reduced fat milk (coefficient = 0.23, 95% CI: 0.07; 0.40) and skim milk (coefficient = 0.28, 95% CI: 0.02; 0.53), reported higher fruit consumption (Table 3).

The mean frequency/number of grain and vegetable intake by different types of long-term milk consumption for males and females are shown in Supplementary Figure S1. Males with soy milk had the highest grain and vegetable intake, while females with reduced fat milk had the highest grain intake. Males with soy milk had significantly higher grain intake than females (*p* < 0.001), while no significant differences were found for grain intake among other types of long-term milk consumption in males and females (Supplementary Figure S1a). Females with long-term whole milk and reduced fat milk had significantly higher fruit intake than males (*p* = 0.01 and *p* < 0.001), while no differences were found for fruit intake among other types of long-term milk consumption in males and females (Supplementary Figure S1b).

### Sensitivity Analysis

We performed a sensitivity analysis with a total of 18,603 participants diagnosed with CVD and with complete baseline data, but without long-term milk behaviour tracking, recording 7308 deaths until 30 September 2018. Significant differences were also found across different types of milk consumption and survival for males and females (both *p* < 0.001), confirming that whole milk had the lowest survival rate for males and females (Supplementary Figure S2, Supplementary Table S1).

Consistent with the main finding, we found that the types of milk consumption were differentially associated with survival in males with IHD (Supplementary Table S2). Compared to whole milk consumption, males (HR = 0.83, 95% CI: 0.72; 0.94) with reduced fat milk consumption had lower risk of mortality for participants with IHD.

However, differently from the main analysis on long-term dairy consumption, in the sensitivity analysis, we also found a benefit of reduced fat milk consumption and IHD in females (Supplementary Table S2) and cerebrovascular disease in males (Supplementary Table S3). After adjustment of co-variates in females with IHD, those with reduced fat milk had lower risk of mortality (HR = 0.66, 95% CI: 0.53; 0.82) than females with whole milk consumption. In males with cerebrovascular disease, those with reduced fat milk consumption had a lower risk of mortality (HR = 0.65, 95% CI: 0.48; 0.88) than males with whole milk consumption.

## 4. Discussion

We found in 7236 males and females with CVD diagnosis presenting with 1101 deaths over 8.4 years that long-term whole milk consumption across two-time points was associated with a higher risk of mortality than reduced fat milk consumption. This was confirmed in 18,603 participants with 7308 deaths with a single time-point report.

Recently there is more consistent evidence pointing towards total dairy intake associated with lower cardiovascular mortality. For example, the Prospective Urban Rural Epidemiology (PURE) study including data from 21 countries found that a higher intake of total dairy and milk associated with lower cardiovascular mortality than a lower dairy and milk intake, whereas a higher intake of cheese had a neutral effect on CVD [11]. A meta-analysis of nine cohort studies reported that a high total milk consumption has no effect on CVD, but was associated with a lower risk of hypertension [29]. Another meta-analysis of 47 prospective cohort studies also concluded that total dairy consumption was significantly associated with a lower risk of cardiovascular mortality [5]. In our study, there was only a small proportion of males and females who reported to have no dairy and cheese consumption (0.74% of males and 1.06% of females for total dairy, 1.9% of males and 1.43% females for cheese), preventing us from performing reliable comparisons with those who did consume dairy. However, our results provided more insightful findings highlighting the roles of different types of long-term milk consumption in relation to mortality among males and females diagnosed with CVD.

We found that long-term whole milk consumption was associated with a higher risk of mortality than other types of long-term milk consumption, in particular reduced fat milk. Although controversial results have been reported across studies regarding whole milk intake, our finding is consistent with the conclusion from several large prospective studies. A recently pooled study including three prospective cohort studies (the Nurses’ Health Study, Nurses’ Health Study II and Health Professionals Follow-up Study), involving 168,153 women and 49,602 men, concluded that high whole milk intake was associated with higher risks of cardiovascular mortality than those with low intake, while no associations were found for other types of milk (reduced fat milk or skim milk) and cardiovascular mortality [4]. This finding could be explained by saturated fatty acids from whole milk affecting the blood lipid profile and promoting atherosclerosis [4]. This explanation is supported by a recent Cochrane review including 15 randomised controlled trials that used a variety of effective interventions to reduce saturated fat in reducing the risk of cardiovascular events [30].

In Western societies, healthy dietary guidelines often encourage people (aged over 2 years) to reduce full-fat and increase low-fat dairy consumption to prevent CVD [1,2,3]. However, based on the existing literature, there is not enough evidence to support reduced fat dairy over whole fat dairy and associated benefits for CVD prevention in the general population (31). Our results support the beneficial role of long-term reduced fat milk consumption in relation to mortality in both males and females diagnosed with CVD when compared to whole milk—aligning with the latest recommendation from the Heart Foundation of Australia [31,32].

Patients who develop CVD are often encouraged to adopt a healthier lifestyle by replacing whole milk with reduced fat milk [31]. We found in our population sample that 1 in 2 (48% of males and 54% of females) people with CVD consume reduced fat milk, and it prevented 31% of deaths for males and 41% of deaths for females than those using long-term whole milk. However, 40% of males and 31% of females with CVD were reported to consume whole milk implying that healthy dairy advice has not made a sufficient impact on behaviours.

In terms of sex differences on food consumption, many studies indicate that differences in dietary habits or patterns may impact health outcomes [33]. However, few studies specifically indicate differences in dairy intake in males and females. The German National Nutrition Survey II found that women aged 35–80 years consumed more dairy than men of the same age [34]. However, research on how it related to health outcomes in males and females needs to be further developed.

With regards to a subgroup analysis in those with specific types of CVD, we found a specific benefit of long-term reduced fat milk in males with IHD. The link between different types of milk and specific CVDs is limited, with the results pointing towards a higher intake of high-fat milk being associated with a higher risk of stroke [35]. Moreover, these studies often draw attention to dairy consumption in the primary prevention of CVD, whereas our study focuses on secondary prevention, providing guidance to patients and clinicians managing diet after a diagnosis of CVD.

Interestingly, we found that people with milk consumption other than whole milk also had a higher intake of grain and fruit potentially constituting an overall healthier diet [36,37]. These findings suggest that people with a “healthy dairy option” (i.e., reduced fat milk) tended to make better healthy food choices than those who often consumed whole milk.

One of the highlights from our study design is that we examined long-term milk consumption. Many prospective cohort studies draw conclusions on different types of dairy in relation to cardiovascular outcomes but often include participants with different dairy consumption behaviours over time and examine its link to cardiovascular events by using different statistical models [8,35], or commonly linking one-time dairy consumption data to cardiovascular events [5]. It is possible that these approaches may lead to controversial and opposing findings.

The strengths of our study are that it involved a large population sample followed over a long period of time, including the tracking of different types of milk consumption by different sexes. Limitations include the use of self-reported data, which may potentially introduce measurement bias. Secondly, the volume of milk intake (i.e., frequencies and serving sizes) was not reported, thereby not allowing us to perform dose-specific analyses. Moreover, a short dietary questionnaire does not capture all relevant food, such as yogurt, not allowing us to adjust for this in the analysis. Some dietary confounders, such as fast-food consumption, were not able to be included in the analysis because of data availability. Thirdly, there were relatively small numbers consuming skim milk and soy milk, and the results on these two specific milk types in relation to mortality are likely underpowered. Fourthly, it might be possible that people change their milk consumption behaviour across two-time points (e.g., consumed whole milk at baseline while other types at follow-up) that may impact mortality. However, we only found a small proportion of people that changed their milk consumption behaviour (N = 370, 5% of entire sample) which prevented us from doing a detailed analysis. Lastly, although it would be valuable to focus specifically on cardiovascular mortality in the analysis, cause of death information was only available until 30 June 2016.

## 5. Conclusions

Our study demonstrates the potential benefits of consuming reduced fat milk over the long term in people with CVD. Compared to people who often consume whole milk, those who often consume reduced fat milk presented with a 31% reduction in all-cause deaths in males, a 37% risk reduction in males with ischemic heart disease and a 41% reduction in females.

## Figures and Tables

**Table 1:** Baseline characteristics by different types of long-term milk consumption for males and females.

	Males				
	Whole Milk	Reduced Fat Milk	Skim Milk	Soy Milk	*p* Value
*N* (%)	931 (40.3)	1111 (48.1)	139 (6.02)	128 (5.54)	
Age (mean, SD)	68.6 (9.63)	67.5 (8.99)	65.7 (8.82)	68.6 (9.61)	0.002
Marital status (*N*, %)					
Married/partner	694 (75.1)	960 (87.1)	115 (84.6)	102 (80.3)	<0.001
Single/divorce/separated/widowed	230 (24.9)	142 (12.9)	21 (15.4)	25 (19.7)	
Education (*N*, %)					
Low	285 (31.4)	259 (23.6)	33 (24.3)	29 (23.0)	<0.001
Medium	453 (49.8)	539 (49.0)	70 (51.5)	64 (50.8)	
High	171 (18.8)	302 (27.5)	33 (24.3)	33 (26.2)	
SEIFA * (*N*, %)					
Low	318 (35.1)	302 (27.9)	39 (28.5)	34 (28.3)	0.004
Medium	311 (34.4)	367 (34.0)	49 (35.8)	49 (40.8)	
High	276 (30.5)	412 (38.1)	49 (35.8)	37 (30.8)	
Smoke (*N*, %)					
No	859 (93.1)	1,075 (97.7)	134 (97.1)	128 (100)	<0.001
Yes	64 (6.93)	25 (2.27)	4 (2.90)	-	
Physical activity ** (*N*, %)					
Inadequate	256 (28.1)	244 (22.1)	31 (22.5)	23 (18.1)	0.005
Adequate	655 (71.9)	859 (77.9)	107 (77.5)	104 (81.9)	
Alcohol, drinks per week (mean, SD)	10.1 (11.5)	8.60 (9.05)	9.84 (10.2)	6.66 (8.27)	<0.001
Vegetables, serves per day (mean, SD)	3.31 (2.76)	3.71 (2.56)	3.71 (2.50)	3.82 (3.24)	0.005
Fruit, serves per day (mean, SD)	1.61 (1.45)	1.90 (1.37)	2.06 (1.63)	2.32 (1.38)	<0.001
Grains, times per week (mean, SD)	4.85 (2.69)	5.59 (2.31)	5.74 (2.14)	6.27 (1.84)	<0.001
Protein, times per week ^¶^ (mean, SD)	3.75 (2.14)	4.09 (2.05)	4.03 (1.91)	3.79 (2.31)	0.002
Survival status					
Alive	737 (79.2)	970 (87.3)	127 (91.4)	112 (87.5)	<0.001
Died	194 (20.8)	141 (12.7)	12 (8.63)	16 (12.5)	
	**Females**				
*N* (%)	358 (31.0)	619 (53.6)	111 (9.62)	66 (5.72)	
Age (mean, SD)	69.3 (11.1)	67.2 (9.70)	65.4 (9.90)	67.7 (8.77)	<0.001
Marital status (*N*, %)					
Married/partner	211 (59.1)	418 (67.9)	84 (75.7)	47 (71.2)	0.003
Single/divorce/separated/widowed	146 (40.9)	198 (32.1)	27 (24.3)	19 (28.8)	
Education (*N*, %)					
Low	184 (53.0)	268 (43.9)	50 (46.3)	23 (36.5)	0.09
Medium	112 (32.3)	228 (37.3)	38 (35.2)	25 (39.7)	
High	51 (14.7)	115 (18.8)	20 (18.5)	15 (23.8)	
SEIFA * (*N*, %)					
Low	154 (44.8)	214 (35.5)	32 (29.6)	25 (38.5)	0.003
Medium	110 (32.0)	178 (29.5)	39 (36.1)	23 (35.4)	
High	80 (23.3)	211 (35.0)	37 (34.3)	17 (26.2)	
Smoke (*N*, %)					
No	316 (89.3)	599 (96.8)	104 (94.6)	66 (100)	<0.001
Yes	38 (10.7)	20 (3.23)	6 (5.45)	-	
Physical activity ** (*N*, %)					
Inadequate	115 (32.6)	157 (25.8)	36 (32.4)	10 (15.4)	0.01
Adequate	238 (67.4)	452 (74.2)	75 (67.6)	55 (84.6)	
Alcohol, drinks per week (mean, SD)	3.81 (6.43)	3.68 (5.26)	3.79 (5.42)	2.89 (4.50)	0.68
Vegetables, serves per day (mean, SD)	4.32 (2.75)	4.56 (2.55)	4.65 (2.24)	4.96 (2.45)	0.21
Fruit, serves per day (mean, SD)	1.80 (1.25)	2.17 (1.34)	2.20 (1.18)	2.23 (1.06)	<0.001
Grains, times per week (mean, SD)	4.63 (2.96)	5.19 (2.50)	5.14 (2.51)	5.27 (2.52)	<0.001
Protein, times per week ^¶^ (mean, SD)	3.87 (2.16)	4.12 (2.05)	4.06 (2.31)	4.03 (2.22)	0.36
Survival status					
Alive	285 (79.6)	560 (90.5)	97 (87.4)	59 (89.4)	<0.001
Died	73 (20.4)	59 (9.53)	14 (12.6)	7 (10.6)	

* Socio-Economic Indexes for Areas (SEIFA) is based on three quantiles (low, medium, high) of Index of Relative Socio-economic Advantage and Disadvantage. ** Adequate physical activity was identified if people spent 150 min of moderate intensity physical activity or 75 min of vigorous intensity physical activity per week. ^¶^ Protein includes lean meat and poultry.

**Table 2:** Cox proportional hazard regression models of the association between different types of long-term milk consumption and survival by males and females ^¶^.

Types of Milk	People with Cardiovascular Disease			
	Crude Model		Adjusted Model *	
	Hazard Ratio (HR)	*p* Value	Hazard Ratio (HR)	*p* Value
	Males			
Whole milk (*N* = 931)	1		1	
Reduced fat milk (*N* = 1111)	**0.58 (0.47; 0.72)**	**<0.001**	**0.69 (0.54; 0.89)**	**0.004**
Skim milk (*N* = 139)	**0.40 (0.22; 0.71)**	**0.002**	0.69 (0.37; 1.27)	0.23
Soy milk (*N* = 128)	**0.55 (0.33; 0.92)**	**0.02**	0.66 (0.38; 1.16)	0.15
	Females			
Whole milk (*N* = 358)	1		1	
Reduced fat milk (*N* = 619)	**0.44 (0.31; 0.62)**	**<0.001**	**0.59 (0.38; 0.91)**	**0.016**
Skim milk (*N* = 111)	0.61 (0.35; 1.08)	0.09	1.21 (0.60; 2.47)	0.60
Soy milk (*N* = 66)	0.53 (0.24; 1.15)	0.11	0.99 (0.44; 2.23)	0.98
	**People with Ischemic Heart Disease**			
	Males			
Whole milk (*N* = 400)	1		1	
Reduced fat milk (*N* = 603)	**0.47 (0.34; 0.66)**	**<0.001**	**0.63 (0.43; 0.92)**	**0.017**
Skim milk (*N* = 79)	**0.39 (0.17; 0.88)**	**0.024**	0.63 (0.25; 1.58)	0.32
Soy milk (*N* = 72)	0.52 (0.25; 1.08)	0.08	0.72 (0.34; 1.53)	0.39
	Females			
Whole milk (*N* = 109)	1		1	
Reduced fat milk (*N* = 249)	**0.41 (0.22; 0.75)**	**0.004**	0.79 (0.36; 1.73)	0.55
Skim milk (*N* = 48)	0.49 (0.18; 1.30)	0.15	1.56 (0.46; 5.37)	0.48
Soy milk (*N* = 28)	0.81 (0.28; 2.37)	0.70	1.88 (0.56; 6.30)	0.31

^¶^ Bold indicates the significant results. * Adjusted for socio-economic status, health behaviours, consumption of other food groups and other chronic conditions.

**Table 3:** The association between different types of long-term milk, grain and fruit intake ^¶^.

	Grain Intake	
	Coefficients *	*p* Value
Males		
Whole milk (*N* = 1862)	0	
Reduced fat milk (*N* = 2222)	**0.62 (0.41; 0.82)**	**<0.001**
Skim milk (*N* = 278)	**0.75 (0.34; 1.16)**	**<0.001**
Soy milk (*N* = 256)	**1.07 (0.64; 1.49)**	**<0.001**
Females		
Whole milk (*N* = 716)	0	
Reduced fat milk (*N* = 1238)	**0.86 (0.54; 1.18)**	**<0.001**
Skim milk (*N* = 222)	**0.74 (0.22; 1.26)**	**0.005**
Soy milk (*N* = 132)	0.40 (−0.23; 1.03)	0.12
	**Fruit Intake**	
Males		
Whole milk (*N* = 1724)	0	
Reduced fat milk (*N* = 2147)	**0.11 (0.001; 0.23)**	**0.048**
Skim milk (*N* = 268)	**0.40 (0.18; 0.63)**	**<0.001**
Soy milk (*N* = 216)	**0.57 (0.34; 0.81)**	**<0.001**
Females		
Whole milk (*N* = 680)	0	
Reduced fat milk (*N* = 1206)	**0.23 (0.07; 0.40)**	**0.004**
Skim milk (*N* = 247)	**0.28 (0.02; 0.53)**	**0.033**
Soy milk (*N* = 126)	0.01 (−0.30; 0.32)	0.96

^¶^ Bold indicates the significant results. * Adjusted for socio-economic status, health behaviours, consumption of other food groups and other chronic condition.

## Data Availability

The 45 and Up Study is managed by the Sax Institute. For data access, please contact the 45 and Up Study team at 45andUp.research@saxinstitute.org.au.

## Supplementary Materials

The following supporting information can be downloaded at: https://www.mdpi.com/article/10.3390/nu14030704/s1, Figure S1: Mean frequency/number of grain and fruit intake by different types of long-term milk consumption for males and females; Figure S2: Kaplan–Meier survival estimates for different types of milk and survival by males and females; Table S1: Cox proportional hazard regression models of different types of milk and survival for males and females with CVD; Table S2: Cox proportional hazard regression models of different types of milk and survival for males and females with ischemic heart disease. Table S3: Cox proportional hazard regression models of different types of milk and survival for males and females with cerebrovascular disease.
