# Supplementary material for: Different Types of Long-Term Milk Consumption and Mortality in Adults with Cardiovascular Disease: A Population-Based Study in 7236 Australian Adults over 8.4 Years
Source: Nutrients. 2022 Feb 8;14(3):704. doi: 10.3390/nu14030704 (PMC8839098; doi:10.3390/nu14030704)
Supplement: Supplementary file 1 [file nutrients-14-00704-s001.zip › nutrients-1579540-supplementary.pdf]

Supplementary Figure S1. Mean frequency/number of grain and fruit intake by different types of long-term milk consumption for males and females

a)

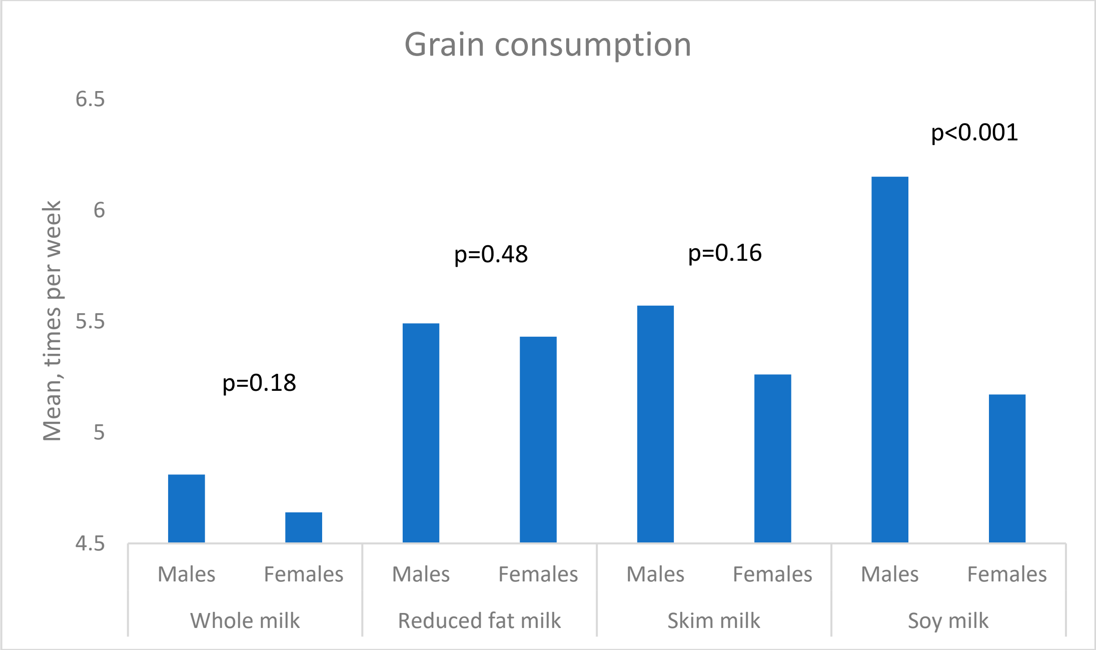

b)

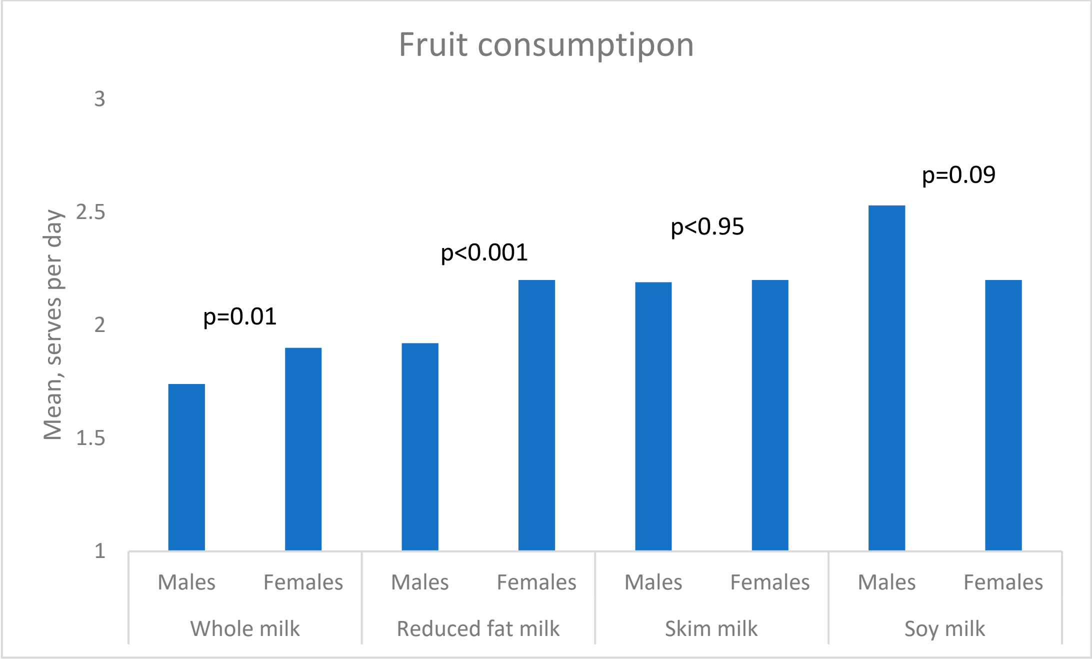

Supplementary Figure S2. Kaplan-Meier survival estimates for different types of milk and survival by males and females

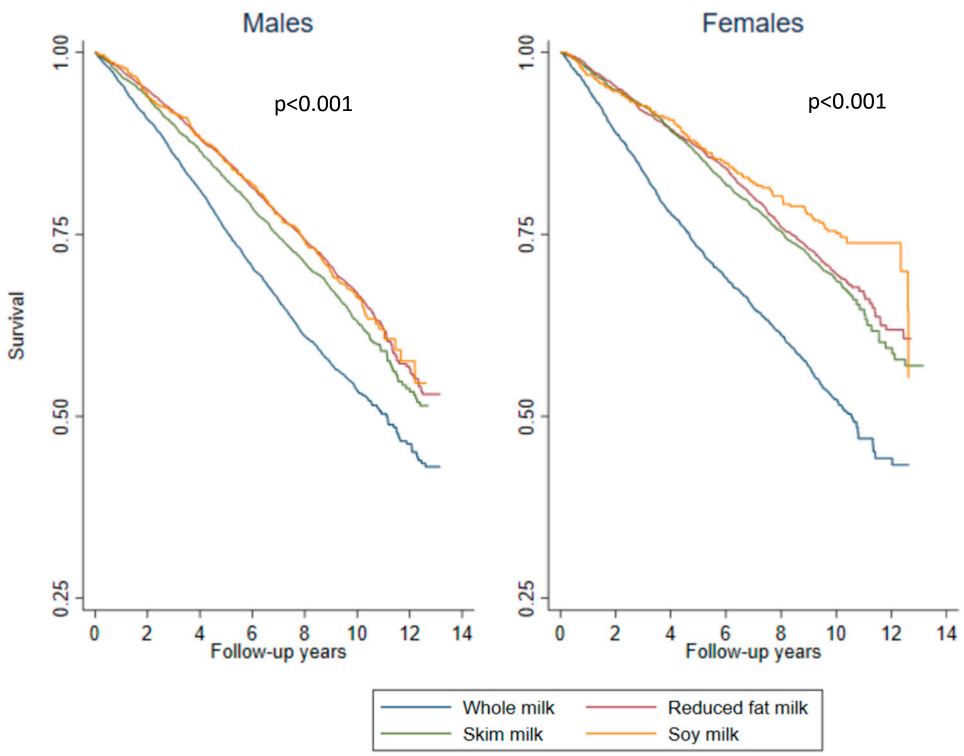

Supplementary Table S1. Cox proportional hazard regression models of different types of milk and survival for males and females with CVD

Types of milk	Crude model		Adjusted model*	
	**Males**			
	**Hazard Ratio**	**P value**	**Hazard Ratio**	**P value**
Whole milk (N=3,612)	1		1	
Reduced fat milk (N=4,098)	**0.65 (0.61; 0.70)**	**<0.001**	**0.81 (0.75; 0.88)**	**<0.001**
Skim milk (N=2,402)	**0.74 (0.68; 0.80)**	**<0.001**	**0.82 (0.75; 0.90)**	**<0.001**
Soy milk (N=581)	**0.66 (0.57; 0.76)**	**<0.001**	**0.76 (0.65; 0.90)**	**0.001**
	**Females**			
Whole milk (N=1,691)	1		1	
Reduced fat milk (N=2,369)	**0.55 (0.49; 0.60)**	**<0.001**	**0.75 (0.67; 0.84)**	**<0.001**
Skim milk (N=1,901)	**0.57 (0.52; 0.63)**	**<0.001**	**0.81 (0.72; 0.92)**	**0.001**
Soy milk (N=355)	**0.44 (0.36; 0.55)**	**<0.001**	**0.69 (0.54; 0.89)**	**0.004**

** Adjusted for socio-economic status, health behaviours, consumption of other food groups and other chronic conditions.*

Supplementary Table S2. Cox proportional hazard regression models of different types of milk and survival for males and females with ischemic heart disease

Types of milk	Crude model		Adjusted model*	
	Hazard Ratio (HR)	P value	Hazard Ratio (HR)	P value
		**Males**		
Whole milk (N=1,430)	1		1	
Reduced fat milk (N=2,069)	**0.67 (0.60; 0.75)**	**<0.001**	**0.83 (0.72; 0.94)**	**0.003**
Skim milk (N=1,219)	**0.70 (0.61; 0.80)**	**<0.001**	**0.77 (0.67; 0.90)**	**0.001**
Soy milk (N=281)	**0.71 (0.57; 0.89)**	**0.003**	0.80 (0.62; 1.04)	0.09
		**Females**		
Whole milk (N=516)	1		1	
Reduced fat milk (N=926)	**0.47 (0.39; 0.56)**	**<0.001**	**0.66 (0.53; 0.82)**	**<0.001**
Skim milk (N=749)	**0.55 (0.46; 0.66)**	**<0.001**	0.84 (0.68; 1.05)	0.13
Soy milk (N=138)	**0.30 (0.19; 0.45)**	**<0.001**	**0.56 (0.34; 0.93)**	**0.023**

** Adjusted for socio-economic status, health behaviours, consumption of other food groups and other chronic conditions.*

Supplementary Table S3. Cox proportional hazard regression models of different types of milk and survival for males and females with cerebrovascular disease

Types of milk	Crude model		Adjusted model*	
	Hazard Ratio (HR)	P value	Hazard Ratio (HR)	P value
		**Males**		
Whole milk (N=284)	1		1	
Reduced fat milk (N=245)	**0.61 (0.48; 0.78)**	**<0.001**	**0.65 (0.48; 0.88)**	**0.005**
Skim milk (N=147)	0.91 (0.70; 1.18)	0.47	0.75 (0.55; 1.03)	0.08
Soy milk (N=30)	0.78 (0.46; 1.32)	0.35	0.60 (0.33; 1.08)	0.09
		**Females**		
Whole milk (N=140)	1		1	
Reduced fat milk (N=157)	**0.55 (0.39; 0.77)**	**0.001**	0.82 (0.54; 1.27)	0.38
Skim milk (N=110)	**0.65 (0.45; 0.94)**	**0.022**	0.96 (0.60; 1.54)	0.86
Soy milk (N=21)	0.71 (0.36; 1.42)	0.33	0.90 (0.37; 2.16)	0.81

** Adjusted for socio-economic status, health behaviours, consumption of other food groups and other chronic conditions.*
